# Impact of Body Mass Index on Plasma N-Terminal ProB-Type Natriuretic Peptides in Chinese Atrial Fibrillation Patients without Heart Failure

**DOI:** 10.1371/journal.pone.0105249

**Published:** 2014-08-21

**Authors:** Li-hui Zheng, Ling-min Wu, Yan Yao, Wen-sheng Chen, Jing-ru Bao, Wen Huang, Rui Shi, Kui-jun Zhang, Shu Zhang

**Affiliations:** State Key Laboratory of Cardiovascular Disease, Clinical EP Lab & Arrhythmia Center, Fuwai Hospital, National Center for Cardiovascular Diseases, Chinese Academy of Medical Sciences and Peking Union Medical College, Beijing, China; University of Catanzaro Magna Graecia, Italy

## Abstract

**Background:**

An inverse relationship between body mass index (BMI) and circulating levels of N-terminal proB-type natriuretic peptide (NT-proBNP) has been demonstrated in subjects with and without heart failure. Obesity also has been linked with increased incidence of atrial fibrillation (AF), but its influence on NT-proBNP concentrations in AF patients remains unclear. This study aimed to investigate the effect of BMI on NT-proBNP levels in AF patients without heart failure.

**Methods:**

A total of 239 consecutive patients with AF undergoing catheter ablation were evaluated. Levels of NT-proBNP and clinical characteristics were compared in overweight or obese (BMI≥25 kg/m^2^) and normal weight (BMI<25 kg/m^2^) patients.

**Results:**

Of 239 patients, 129 (54%) were overweight or obese. Overweight or obese patients were younger, more likely to have a history of nonparoxysmal AF, hypertension, and diabetes mellitus. Levels of NT-proBNP were significantly lower in overweight or obese than in normal weight subjects (P<0.05). The relationship of obesity and decreased NT-proBNP levels persisted in subgroup of hypertension, both gender and both age levels (≥65 yrs and <65 yrs).Multivariate linear regression identified BMI as an independent negative correlate of LogNT-proBNP level.

**Conclusions:**

An inverse relationship between BMI and plasma NT-proBNP concentrations have been demonstrated in AF patients without heart failure. Overweight or obese patients with AF appear to have lower NT-proBNP levels than normal weight patients.

## Introduction

B-type natriuretic peptide (BNP) is synthesized as preproBNP in response to the stretch and pressure overload of the cardiac myocyte. After enzymatic cleavage, it is released into the circulation system in equimolar proportions as the hormonally active BNP and the inactive N-terminal fragment (N-terminal-pro-B-type natriuretic peptide, NT-proBNP).The expression of NT-proBNP is affected by several variations, such as age, gender, hypertension, renal function and thyroid function [1].

Several recent reports suggest that obesity, as indexed by elevated body mass index (BMI), may also affect NT-proBNP levels, with lower circulating levels in those with higher BMI in subjects with acute or chronic heart failure [2], [3], significant coronary artery disease or acute myocardial infarction [4], [5] and healthy general populations [6]. However, isolated study showed that obesity is not statistically associated with NT-proBNP in asymptomatic patients with hypertension [7].

Atrial fibrillation (AF) is the most common cardiac arrhythmia in the clinical practice. NT-proBNP levels are increased in AF [8], and have proven their potential utility in the risk stratification, prognostication, and therapeutic decision-making in AF [9]–[11]. However, the effect of obesity on NT-proBNP in AF patients has yet to be clarified. We aimed to explore this relationship in the present study. Because AF has been linked with congestive heart failure, we hypothesized that a similar relationship might exist in AF.

## Methods

### Patients

Two hundred and thirty-nine consecutive patients with AF undergoing radiofrequency catheter ablation in our institution between January 2007 and January 2009 were included in this study. Exclusion criteria included chronic heart failure or left ventricular ejection fraction (LVEF) ≤50%, cardiomyopathy, valvular heart disease, hepatic or renal failure, acute coronary syndrome, acute pulmonary embolism, chronic obstructive pulmonary disease, rheumatic heart disease, and thyroid dysfunction. Informed written consent was obtained from all patients, and this study was approved by the Ethics Committee of Fuwai Hospital and clinical investigations are conducted according to the principles expressed in the Declaration of Helsinki.

### Clinical characteristics

Patients were interviewed and records were reviewed to determine past medical history, medications, and pertinent laboratory values. Left atrial diameter (LAD), left ventricular end diastolic diameter (LVEDD), and LVEF was determined within 3 days before the ablation procedure by echocardiography. Blood pressure and heart rate were measured in the morning upon admission, prior to the procedure. The BMI was calculated as body weight (kg) divided by the square of the height (m) at the time of the admission.

According to the World Health Organization/National Institutes of Health classification scheme, BMI groups were divided into 2 categories: overweight or obesity (BMI≥25 kg/m^2^) and normal weight (BMI<25 kg/m^2^) [12].

### Measurement of NT-proBNP concentrations

Venous blood samples were collected from the antecubital vein in the morning upon admission, prior to the procedure. For each NT-proBNP measurement, 5 ml of whole blood was collected into tubes containing potassium EDTA (1 mg/ml blood) as anticoagulant to produce plasma. Specimens were centrifuged at 3000 r/min within 1 hour of collection. The resulting serum or plasma was aliquoted into respective storage vials and then frozen and maintained at −70°C until analysis. All testing and system operation were in accordance with manufacturer's recommendations. The serum specimen was used for NT-proBNP measurements using the assay kits (BIOMEDICA Medizinpordukte GmbH & Co KG, Wein, Australia). Assays were performed in a single run and normalized to a standard curve. Inter-assay coefficients of variation for NT-proBNP were <9%.

### Statistical analysis

All statistical analyses were performed in SPSS 13.0 (SPSS, Inc., Chicago, Illinois). Continuous data are presented as mean ± SD or median plus interquartile ranges, as appropriate. Because of the large range in NT-proBNP, analysis and results of its log are also reported. Categorical variables were compared using the Pearson's χ^2^ test. With continuous variables, group mean values were compared using the Student *t* test or analysis of variants, as appropriate. If the data distribution did not follow the normality assumption, the Wilcoxon rank-sum test was used. P<0.05 is considered statistically significant.

Further linear regression was performed to determine the independent relationship of BMI and LogNT-proBNP. In addition to BMI, the following covariates were also analyzed by multivariate linear regression: age, gender, AF duration, nonparoxysmal AF, smoking, diabetes mellitus, hypertension, coronary heart disease, serum creatinine, systolic blood pressure, diastolic blood pressure, LAD, LVEDD, LVEF, heart rate, using of warfarin, aspirin, angiotensin-converting enzyme inhibitor (ACEI)/angiotensin receptor blocker (ARB), statin, calcium channel block,βblocker, propafenone, and amiodarone. The models were fit by a stepwise backward-selection algorithm, where all variables were entered into the original model and then variables with probability values of >0.05 were removed.

## Results

### Patients

The study included 239 consecutive patients (age 56±11 yrs; 75% men) with AF. Of these, the mean LVEF was 62±7%, the mean LAD was 41±8 mm, and the mean LVEDD was 50±4 mm. The mean serum creatinine was 81±14 umol/L. Of these, 121 (50.6%) had hypertension, 27 (11.3%) had diabetes mellitus, and 11(4.6%) had a history of coronary heart disease. The total population's BMI was 24.7±3.0 kg/m^2^, 129 (54%) were overweight or obese (≥25 kg/m^2^), and 110 (46%) normal weight (<25 kg/m^2^). Overweight or obese patients were younger, and more likely to have a history of nonparoxysmal AF, hypertension, and diabetes mellitus (Table 1).

**Table 1:** Characteristics of patients with body mass index (BMI)≥25 or <25 kg/m^2^.

	Overweight or obese: BMI≥25 kg/m^2^ (n = 129)	Normal weight: BMI<25 kg/m^2^ (n = 110)	P value
BMI(kg/m^2^)	26.9±1.9	22.0±1.6	<0.05
Age (yrs)	55±12	58±9	<0.05
Men (%)	94/129(72.9%)	85/110(77.3%)	NS
AF duration (yrs)	6.4±7.1	6.7±5.6	NS
Nonparoxysmal AF	50/129(38.8%)	29/110(26.4%)	<0.05
Smoking	50/129(38.8%)	41/110(37.2%)	NS
Diabetes mellitus(%)	20/129(15.5%)	7/110(6.4%)	<0.05
Hypertension(%)	76/129(58.9%)	45/110(40.9%)	<0.05
CHD(%)	6/129(4.7%)	5/110(4.5%)	NS
Serum creatinine (umol/L)	81±15	81±13	NS
Systolic blood pressure (mmHg)	130±16	126±15	NS
Diastolic blood pressure (mmHg)	79±13	77±11	NS
Heart rate (bpm)	79±18	76±15	NS
LAD (mm)	43±7	41±8	<0.05
LVEDD (mm)	50±4	49±5	<0.05
LVEF (%)	62±7	62±7	NS
Medications			
Warfarin(%)	6/129(4.7%)	4/110(3.6%)	NS
Aspirin(%)	24/129(18.6%)	21/110(19.1%)	NS
ACEI/ARB(%)	47/129(36.4%)	24/110(21.8%)	<0.05
Statin(%)	9/129(7.0%)	6/110(5.5%)	NS
CCB(%)	16/129(12.4%)	5/110(4.5%)	<0.05
βblocker(%)	42/129(32.6%)	33/110(30%)	NS
Profenanone(%)	19/129(14.7%)	17/110(15.5%)	NS
Amiodarone(%)	23/129(17.8%)	18/110(16.4%)	NS
NT-proBNP(fmol/ml)	437(324–550)	501(447–638)	<0.05
Log NT-proBNP	2.62±0.18	2.75±0.15	<0.001

CHD = Coronary heart disease; LAD = Left atrial diameter; LVEDD = Left ventricular end diastolic diameter; LVEF =  Left ventricular ejection fraction; ACEI = Angiotensin-converting enzyme inhibitor; ARB =  Angiotensin receptor blocker; CCB =  Calcium channel blocker; NT-proBNP = N-terminal proB-type natriuretic peptide; NS = No significant.

### Comparison of NT-proBNP levels in overweight or obese and normal weight patients

**Figure 1:**
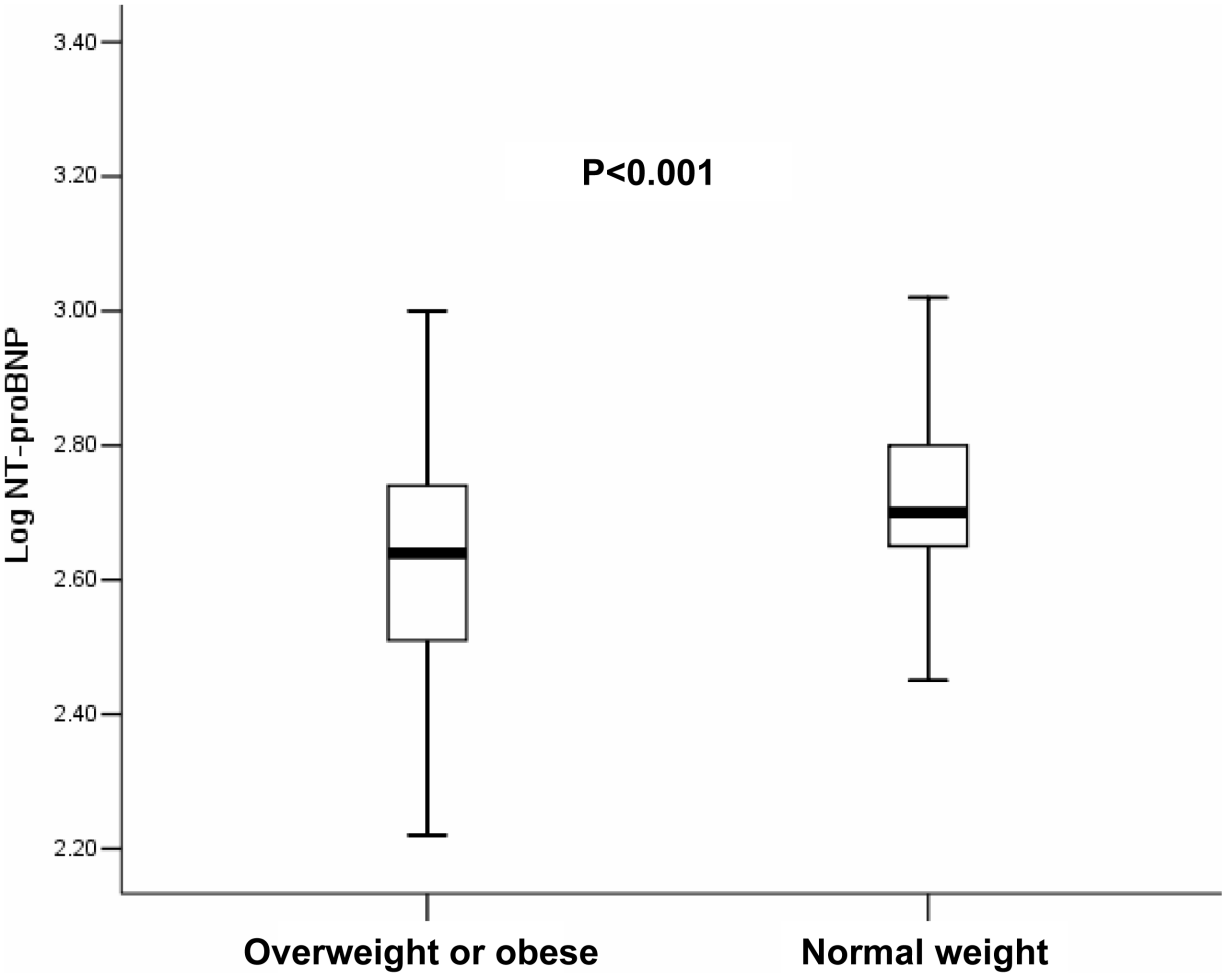
Comparison of LogNT-proBNP between overweight or obese patients and normal weight patients. Box plots of log of circulating N-terminal proB-type natriuretic peptide (NT-proBNP) levels showing medians with interquartile ranges. Overweight or obese patients have lower levels of Log NT-proBNP (2.62±0.18) compared with that of normal weight patients (2.75±0.15) with atrial fibrillation (P<0.001).

Levels of NT-proBNP were significantly lower in overweight or obese patients than in normal weight patients (Table 1) (Figure 1). The median NT-proBNP in overweight or obese patients were 437 fmol/ml, compared to 501 fmol/ml in the normal weight patients. The higher concentrations occurred despite similar LVEF and higher LVEDD and LAD.

To further evaluate the influence of age and its possible interaction with NT-proBNP levels in the context of obesity, we divided the cohort into elderly (≥65 yrs) and nonelderly (<65 yrs), and found that the relationship of obesity and decreased NT-proBNP levels persisted in both nonelderly and elderly (P<0.05), respectively. Similarly, the relationship of obesity and decreased NT-BNP concentrations persisted in patients with hypertension (P<0.001), and over the different gender (P<0.05), respectively (Table 2).

**Table 2:** Relationships of BMI and NT-proBNP levels in the subgroups.

	Overweight or obese: BMI≥25 kg/m^2^ (fmol/ml)	Normal weight: BMI<25 kg/m^2^ (fmol/ml)	P value
**Male (n = 179)**			
NT-proBNP	431(316–552)	489(436–638)	<0.001
Log NT-proBNP	2.69±0.18	2.74±0.15	<0.001
**Female(n = 60)**			
NT-proBNP	436(346–549)	513(468–686)	0.004
Log NT-proBNP	2.63±0.19	2.77±0.16	0.003
**Age<65 yrs(n = 188)**			
NT-proBNP	417(314–537)	495(437–603)	<0.001
Log NT-proBNP	2.60±0.17	2.72±0.13	0.005
**Age≥65 yrs(n = 51)**			
NT-proBNP	490(363–661)	795(490–1097)	0.003
Log NT-proBNP	2.69±0.20	2.88±0.18	0.001
**Hypertension(n = 121)**			
NT-proBNP	437(306–543)	490(447–617)	0.001
Log NT-proBNP	2.62±0.19	2.75±0.15	<0.001

NT-proBNP = N-terminal proB-type natriuretic peptide; BMI = body mass index.

**Figure 2:**
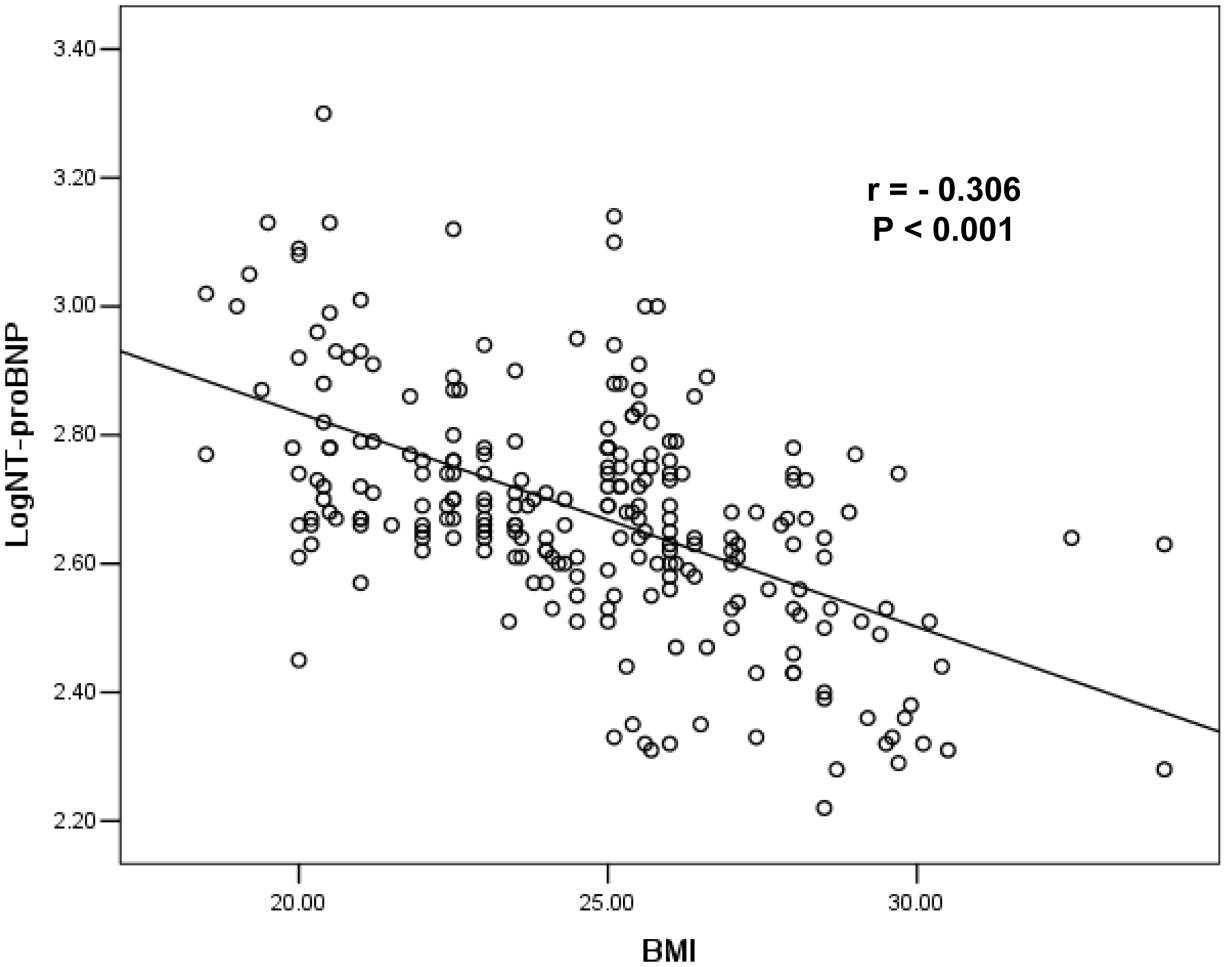
Inverse relationship between LogNT-proBNP and BMI in atrial fibrillation. This figure depicts the raw data demonstrating a significant inverse correlation between log of circulating N-terminal proB-type natriuretic peptide (NT-proBNP) levels and body mass index (BMI) in patients with atrial fibrillation (r = −0.306, P<0.001).

An inverse relationship between the LogNT-proBNP and BMI was found in the total study population (r = −0.306, P<0.001) (Figure 2), and for the two BMI groups, respectively (normal weight r = −0.276, P<0.001; overweigh or obese r = −0.273, P<0.001) and for both genders, respectively (male r = −0.275, P<0.001; female r = −0.381; P<0.001).

### Multivariate predictors of NT-proBNP levels

Multivariate regressions analysis revealed BMI as an independent negative correlate of LogNT-BNP level (t = −10.063, P<0.001). Other independent factors included age (t = 3.415, P = 0.001),using of ACEI/ARB (t = −2.080, P = 0.039), and LAD (t = 7.098, P<0.001). Particularly, we did not find any significant independent influence of hypertension and diabetes mellitus on NT-proBNP levels (Table 3).

**Table 3:** Multivariate linear regression results for detecting independent factors on log-transformed plasma NT-proBNP in AF patients.

Variable	t	P value
Age	3.415	0.001
Gender	1.763	0.079
AF duration	0.721	0.471
Nonparoxysmal AF	0.026	0.979
Smoking	1.559	0.120
Diabetes mellitus	0.987	0.325
Hypertension	0.221	0.826
CHD	0.062	0.951
Serum creatinine	0.810	0.419
Systolic blood pressure	−0.149	0.881
Diastolic blood pressure	1.546	0.124
LAD	7.098	0.000
LVEDD	0.938	0.349
LVEF	−0.423	0.672
Heart rate	−0.358	0.720
Warfarin	0.234	0.815
Aspirin	−0.785	0.433
ACEI/ARB	−2.080	0.039
Statin	1.045	0.297
CCB	1.801	0.069
βblocker	0.916	0.361
Profenanone	0.290	0.772
Amiodarone	1.132	0.259
BMI	−10.063	0.000

NT-proBNP = N-terminal proB-type natriuretic peptide; AF =  Atrial fibrillation; CHD = Coronary heart disease; LAD = Left atrial diameter; LVEDD = Left ventricular end diastolic diameter; LVEF =  Left ventricular ejection fraction; ACEI = Angiotensin-converting enzyme inhibitor; ARB =  Angiotensin receptor blocker; CCB =  Calcium channel blocker; BMI = body mass index.

## Discussion

### Major Findings

This study demonstrates an inverse relationship between higher BMI and lower NT-proBNP levels in AF patients; overweight or obese patients with AF have lower NT-proBNP levels compared to normal weight AF subjects. The validity of this observation is supported by its consistency in subgroup of hypertension, both gender and both age levels (≥65 yrs and <65 yrs).

### Average level and distribution of BMI

In this study, the average level (around 24.7 kg/m^2^) and distribution of BMI was comparable with other Asian studies [4], [13], but it was much lower in comparison with the western populations (around 28 kg/m^2^) [2], [3], [5], [7]. Of this study populations, 46% was normal weight (BMI<25 kg/m^2^), 51% overweight (BMI = 25–29.9 kg/m^2^), and 3% obese (BMI≥30 kg/m^2^). Therefore, in the present study, the actual obese patients were rare, and we divided the study patients into overweight or obese group and normal weight group. The difference of average level and distribution of BMI may be due to an ethnic difference.

### AF and NT-proBNP

Recently, the relationship between AF and NT-proBNP has been intensely studied. NT-proBNP is increased in AF [8]. In a community-based population, increased NT-proBNP levels at baseline independently predicted newly detected AF [8]. Deftereos et al demonstrated that NT-proBNP levels could predict the presence of left atrial thrombus in AF patients of unknown onset and no heart failure [10]. Furthermore, baseline NT-proBNP levels could predict risk of new-onset AF after cardiac surgery [11] or non-cardiac surgery [14], and predict AF recurrence after successful electrical cardioversion [15] or catheter ablation [9]. Thus, NT-proBNP has proven their diagnostic usefulness in the risk stratification, prognostication, and guiding therapy in patients with AF.

### Obesity and NT-proBNP

Many studies have evaluated the relationship between plasma NT-proBNP and obesity. An inverse relationship between BMI and NT-proBNP levels has been demonstrated in subjects with acute or chronic heart failure [2], [3], significant coronary artery disease or acute myocardial infarction [4], [5] and healthy general populations in Framingham Heart study [6]. However, in asymptomatic patients with essential hypertension, with or without left ventricular hypertrophy, the inverse association was not observed [7]. In addition, two recent studies showed that NT-proBNP is closely associated with lean mass than with fat mass [6], and is related with visceral adipose tissue than subcutaneous adipose tissue [16]. Nevertheless, the relationship between plasma NT-proBNP levels and BMI in AF patients has not been analyzed. In one study from the Framingham Heart study [17], Wang et al reported that higher BMI was associated with decreased BNP and N-terminal proatrial natriuretic peptides (NT-proANP) levels. In that study, only a small number of AF patients were included, and accounted for 0.5%, 1% and 2% of the normal, overweight and obese group. Furthermore, the BNP levels of AF patients were not compared among different BMI groups.

In this study, although the patient average BMI level was much lower than the general western populations, the association between higher BMI and lower NT-proBNP levels were observed in AF patients. Furthermore, the validity of our findings is supported by its consistency across both genders, by the adjustment methods with numerous covariates. The present results therefore extend previous studies from heart failure, coronary artery disease to atrial fibrillation, at least in selected patients.

The exact mechanisms for the inverse relationship between NT-proBNP levels and BMI remain uncertain. Natriuretic peptide clearance receptors (NPR-C) are abundant in adipose tissue, and it is suggested that lipolysis is driven partly by natriuretic peptides, leading to the lower levels of natriuretic peptides in obese individuals [18]. However, the structure of NT-proBNP is distinct from natriuretic peptides and thus unlikely to be cleared via NPR-C, suggesting that the inverse relationship may be due to non-clearance mechanisms. A previous study reported that NT-proBNP decreased with increasing BMI in a manner nearly parallel to the decrease of BNP [3]. In addition, in the Framingham Heart study, NT-proANP levels were also lower in the obese subjects [17]. Because both NT-proANP and NT-proBNP are not cleared by NPR-C in adipose tissue, thus, decreased release or impaired synthesis of natriuretic peptides from the heart may at least partly underlie the possible mechanism. In addition, natriuretic peptides itself may exert potential lipolytic effects in human adipose tissue, leading to lipolysis and weight loss [19]. Further studies are required to explore the associated mechanisms.

### Pathophysiological implications

Several studies have demonstrated that obesity is a major risk factor for the development of AF [20], [21]. The potential mechanism may be that eccentric and concentric left ventricular hypertrophy with resultant progressive atrial enlargement [22], [23]. The elevated circulating volume and enhanced neurohormonal activation (obliged by a larger body mass) may also contribute to the left atrium dilation and electrical instability [21], [24]. Although the exact significance of decreased NT-proBNP levels in overweight or obese state of AF patients are not yet understood, it at least raises the possibility that there may be lesser natriuretic-mediated vasodilation and antagonism of the rennin-angiotensin system, or loss of natriuretic ability in overweight or obese patients, which may also play a role in the development of the AF in overweight or obese patients.

### Study limitations

The present study has several limitations. First, this study included Chinese populations, which may be different with those from western countries. As discussed previously, the average level and distribution of BMI in the present study was much lower than that reported in previous western studies. In addition, the use of warfarin for stroke prevention among AF patients was low in this study, although it is similar to other Chinese study [25]. This may reflect the current medical system pattern in China. Thus, our results must be extended to other populations with caution.

Second, this study involved a population of patients referred for catheter ablation of AF.Thus, due to the patients not eligible for ablation have been excluded, therefore, the conclusions may be possibly not extrapolated to the general population of patients with AF.However, with the increasing numbers of patients receiving ablation, we believe that the results of this study remains valuable, at least in selected patients, which could be millions.

Third, the usual dosage of NT-proBNP is based on pg/ml. In this study, the plasma NT-proBNP level was quantitatively determined using competitive enzyme immunoassay from BIOMEDICA; results are given in fmol/ml. In order to convert the results of NT-proBNP from fmol/ml to pg/ml, results should be multiplied by 8.457 [26]. However, we think that the results using fmol/ml did not affect our conclusions between BMI and NT-proBNP.

## Conclusions

In the present study, we demonstrated an inverse association between higher BMI and lower NT-proBNP levels in AF patients. Overweigh or obese patients have reduced levels of NT-proBNP compared to lean patients. In the clinical practice, it should be considered the effect of the obesity on the NT-proBNP levels for the AF patients.
